# Adipose-derived mesenchymal stem cells-derived exosomes containing nano-pearl powder water-soluble matrix promote osteogenic differentiation of MC3T3-E1 cells

**DOI:** 10.1080/15476278.2026.2630547

**Published:** 2026-02-26

**Authors:** Ling Chen, QiuHua Mao, WenBai Zhang, YaNan Cheng

**Affiliations:** a Department of Pediatric Dentistry, Haikou Affiliated Hospital of Central South University Xiangya School of Medicine, Haikou, Hainan Province, P.R. China; b Department of Oral Implantation, Haikou Affiliated Hospital of Central South University Xiangya School of Medicine, Haikou, Hainan Province, P.R. China

**Keywords:** NPP-WSM, ADSC-Exos, osteogenic differentiation

## Abstract

**Objective:**

To explore the synergistic effect of nano-pearl powder (NPP) and adipose-derived stem cell exosomes (ADSC-Exos) on the osteogenic potential of MC3T3-E1 cells.

**Methods:**

The water-soluble matrix of NPP (NPP-WSM) was extracted via freeze-drying, and ADSC-Exos were isolated by ultracentrifugation. NPP-WSM was incorporated into ADSC-Exos through co-incubation to generate NPP-WSM-Exos. MC3T3-E1 cells were treated with NPP-WSM or NPP-WSM-Exos. Cell proliferation and migration were evaluated using CCK-8 and wound-healing assays, respectively. Osteogenic differentiation was assessed by Alizarin Red S staining and alkaline phosphatase (ALP) activity. The expression of osteogenesis-related genes (COL1A1, RUNX2, OCN, and OPN) was measured by qPCR and Western blotting. Transcriptome sequencing (RNA-seq) was conducted to identify signaling pathways activated by NPP-WSM-Exos.

**Results:**

NPP-WSM-Exos displayed distinct exosome morphology and biomarkers, confirming their successful preparation. Significantly, NPP-WSM-Exos enhanced the viability of MC3T3-E1 cells compared to NPP-WSM alone and upregulated the expression of osteogenic genes, including COL1A1, RUNX2, OCN, and OPN, at both the transcriptional and translational levels. Additionally, NPP-WSM-Exos strongly promoted mineralization, as evidenced by the increased calcification observed through Alizarin Red S staining, and elevated alkaline phosphatase (ALP) activity, indicating excellent potential for osteogenic differentiation. Transcriptome sequencing showed that NPP-WSM-Exos significantly enhanced the PI3K/AKT pathway in MC3T3-E1 cells, while protein level detection indicated that NPP-WSM-Exos could increase AKT phosphorylation levels and inhibit GSK3β activity to improve osteogenic efficiency.

**Conclusion:**

The use of adipose-derived stem cell exosomes to encapsulate NPP-WSM can increase the utilization of WSM, promote the proliferation of MC3T3-E1, and enhance the osteogenic differentiation ability.

## Introduction

Bone defects induced by trauma, infection, tumors, or congenital disorders pose a significant global health challenge, necessitating surgical interventions for millions of patients annually.^1^^,^^2^ Conventional clinical approaches, including autografts and allografts, are hindered by challenges such as limited availability of donor tissues, the risk of immune rejection, and potential disease transmission.^3^^,^^4^ Synthetic biomaterials, such as hydroxyapatite and titanium alloys, often exhibit limitations, including inadequate biodegradability, restricted osteoinductivity,^5^ and mechanical incompatibility with native bone tissue.

Therefore, bone tissue engineering (BTE) has attracted much attention as a feasible alternative, which uses bioactive scaffolds to mimic the extracellular matrix (ECM) and promote osteogenic activity.^6^ In recent years, nanoscale materials engineering and drug delivery strategies have provided a more controllable design framework for the functionalization of BTE scaffolds: for example, drug-loaded nanofibers can be parametrically controlled in terms of fiber diameter, pore structure, mechanical properties and degradation behavior through preparation methods such as electrospinning, thereby combining the biomimetic structure of ECM with local and continuous drug release capabilities, providing ideas for integrated “scaffold–drug” therapy.^7^ In addition, inorganic nanoplatforms represented by green synthesis (such as selenium nanoparticles) have been widely discussed for anti-oxidation, anti-inflammation and other biomedical applications due to their tunable particle size/surface chemical characteristics and good biocompatibility, and have shown potential as functional delivery carriers or synergistic therapeutic components.^8^ Among many innovative materials, nano pearl powder (NPP) stands out. It is a bio-derived nanomaterial synthesized by micronizing natural pearl or pearl layer. NPP is mainly composed of aragonite calcium carbonate nanocrystals (CaCO₃, >95%) and contains trace amounts of organic matrix, including proteins, polysaccharides and glycoproteins.^9^ The unique nanostructure of NPP, typically ranging from 50 to 200 nm in diameter, enhances bioavailability and osseointegration compared to traditional pearl formulations.

The water-soluble matrix derived from nano-pearl powder (NPP-WSM), obtained via freeze-drying extraction, encapsulates the bioactive organic fraction solubilized from NPP.^10^ This matrix retains essential osteogenic components, such as soluble calcium ions, collagen-derived peptides, and signaling glycoproteins, while eliminating the crystalline mineral phase.^11^ Notably, NPP-WSM demonstrates enhanced cellular internalization efficiency and controllable release kinetics of bioactive moieties, positioning it as a promising candidate for applications in bone tissue engineering and regenerative therapies.

Furthermore, mesenchymal stem cell-derived exosomes (MSC-Exos) have emerged as transformative vehicles for bone regeneration due to their low immunogenicity and targeted delivery capabilities.^12-15^ The cargo of MSC-Exos, which includes microRNA-21 (miR-21) and bone morphogenetic protein-2 (BMP-2), synergistically activates the ERK1/2 and BMP/Smad signaling pathways, leading to significant enhancements in osteogenic differentiation (exhibiting a 2.1-fold increase in alkaline phosphatase activity) and angiogenesis (demonstrating a 178% increase in endothelial tube formation). Recent advancements in this field feature engineered modifications, such as aptamer targeting, aimed at optimizing homing efficiency.^16^ Gelatin methacryloyl (GelMA) hydrogels facilitate the sustained release of these exosomes (over 80% within 8 d), effectively addressing the challenge of their limited in vivo half-life.^17^^,^^18^

To date, no studies have combined MSC-Exos with NPP-WSM to formulate a composite material for bone repair. Therefore, the objective of this project is to integrate NPP-WSM with umbilical cord mesenchymal stem cells exosomes (ADSCs-Exos) to develop a novel composite material for bone repair, thereby investigating its ability to promote the osteogenic potential of MC3T3-E1 cells at the cellular level.

## Methods

### Preparation of nano-pearl powder water-soluble matrix (NPP-WSM)

NPP was mixed with ultrapure water at a ratio of 1:20 (w/v) at 4 °C and stirred continuously for 48 h to ensure thorough mixing. The resulting mixture was centrifuged at 12,000 × *g* for 30 min. The supernatant was then ultrafiltered using a 3 kDa molecular weight cutoff membrane and lyophilized to obtain NPP-WSM. The NPP-WSM was stored at −80 °C until further use.

### Preparation of umbilical cord mesenchymal stem cell exosomes

ADSCs (purchased from Auris Biotech (Shanghai) Co., Ltd.) were cultured in complete medium supplemented with exosome-free serum for 48 h. The cell supernatant was collected, and the exosomes were isolated using differential ultracentrifugation.

The supernatant underwent initial centrifugation at 300 × *g* for 10 min to remove cells and debris, followed by 2000 × *g* for 20 min and 10,000 × *g* for 30 min. Exosomes were then pelleted at 100,000 × *g* for 70 min using an Optima XE ultracentrifuge (Beckman Coulter) and washed with phosphate-buffered saline (PBS) to eliminate contaminants.

Further purification was achieved through sucrose density gradient centrifugation at 200,000 × *g* for 16 h. Exosomes were characterized by nanoparticle tracking analysis (30–200 nm peak), transmission electron microscopy (TEM), and Western blotting (TSG101+/calnexin−).

### Preparation of NPP-WSM-Exos

NPP-WSM was dissolved in PBS to achieve a concentration of 1 mg/ml. This solution was then combined with the exosome storage solution and allowed to incubate at room temperature for 30 min. The exosomes were subsequently purified once more through ultracentrifugation, and their characteristics were confirmed using electron microscopy and nanoparticle tracking analysis (NTA).

### CCK8 assay for cell viability

MC3T3-E1 cells were cultured in 96-well plates at a density of 5 × 10³ cells per well and treated with NPP-WSM for 48 h. Following treatment, the cell culture medium was removed, and the cells were washed with PBS. Subsequently, CCK-8 solution was added to the cell culture medium at a ratio of 1:9, and the cells were incubated at 37 °C for 2 h. The absorbance readings were taken at 450 nm, with a reference wavelength of 650 nm. Cell viability is expressed as a percentage relative to the untreated control, designated as 100%.

### Flow cytometry to measure cell apoptosis

MC3T3-E1 cells were plated at a density of 5 × 10⁶ cells in a 6-well plate and cultured under standard conditions or stimulated with NPP-WSM or various concentrations of NPP-WSM-EOXs. Following 48 h of treatment, the cells were washed with PBS. They were then trypsinized and resuspended in 2 ml of complete culture medium. The suspension underwent centrifugation at 1000 rpm for 5 min, after which the supernatant was removed to obtain the pellet. The cell pellet was subsequently washed with PBS and resuspended in staining buffer to achieve a cell density of 1 × 10⁶ cells/ml. A volume of 100 μl of the cell suspension was transferred to a flow cytometry tube, followed by the addition of 5 μl each of FITC-Annexin V and propidium iodide. The cells were incubated at room temperature in the dark for 15 min. Finally, 400 μl of staining buffer was added to each tube, and apoptosis analysis was conducted using a flow cytometer.

### Western blot

Cell samples were lysed on ice for 30 min using RIPA buffer. The lysate was then centrifuged at 12,000 × *g* for 15 min at 4 °C, and the supernatant containing the proteins was collected. The protein concentration was quantified using a BCA assay kit, followed by denaturation in 5× loading buffer at 95 °C for 10 min. The proteins were separated using SDS–PAGE gels and subsequently transferred to PVDF membranes via semi-dry electroblotting. The membranes were blocked with 5% nonfat milk and incubated overnight at 4 °C with primary antibodies (COL1A1, RUNX2, OCN, OPN, and GAPDH; at a dilution of 1:1000). After being washed with TBST, the membranes were incubated with HRP-conjugated secondary antibodies (diluted 1:10,000) for 1 h at room temperature. The protein bands were visualized using ECL substrate, and the grayscale values of the target bands were analyzed with Image J software, normalizing them to the grayscale value of GAPDH.

### Alizarin Red S staining to analyze the degree of calcification

MC3T3-E1 cells were induced to differentiate into osteoblasts using an osteogenic differentiation induction medium. The control group did not receive any additional treatment, whereas the treated groups were stimulated with NPP-WSM or varying concentrations of NPP-WSM-Exos. On days 3, 7, and 14, the MC3T3-E1 cells were fixed using a cell fixative for 30 min, rinsed 3× with PBS, and stained with Alizarin Red S solution. Following thorough rinsing with distilled water, the cells were photographed. The images were then quantitatively analyzed using Image J.

### Alkaline phosphatase activity assay

Alkaline phosphatase (ALP) activity was assessed using p-nitrophenyl phosphate (pNPP) as the substrate. The cells were lysed with 0.1% Triton X-100, and the resulting supernatant was incubated with 20 mM pNPP in 1.5 M Tris–MgCl₂ buffer (pH 10.3) at 37 °C for 15 min. The reaction was then halted with 2 M NaOH, and the absorbance was measured at 405 nm. ALP activity was calculated as the amount of p-nitrophenol produced per minute (μmol/min/mg protein), using a standard curve, and was normalized to the total protein concentration determined by the BCA assay.

### Statistical analysis

Each experiment was repeated three times independently, and the experimental results were statistically analyzed using the independent sample *t*-test using IBM SPSS Statistics. *p* value <0.05 was considered to indicate statistical significance.

## Results

### Preparation and characterization of NPP-WSM-Exos

**Figure 1.**
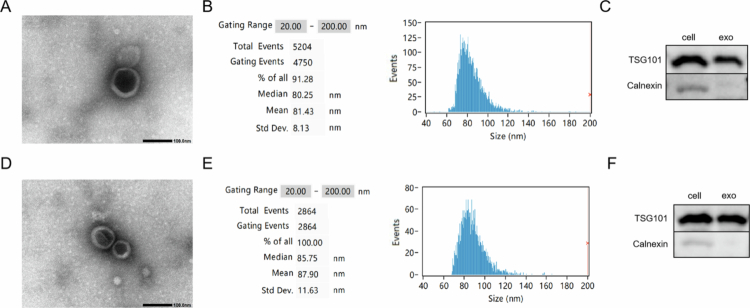
(A) Transmission electron microscopy observation of UCMSCs exosome morphology. (B) Nanoflow cytometry detection of UCMSCs exosome size and concentration. (C) WB detection of UCMSCs exosome protein markers. (D) Transmission electron microscopy observation of NPP-WSM-Exos morphology. (E) Nanoflow cytometry detection of NPP-WSM-Exos size and concentration. (F) WB detection of NPP-WSM-Exos protein markers.

The isolated ADSCs-Exos were characterized in detail. As illustrated in Figure 1A, ADSC-Exos exhibited a typical cup-shaped morphology with a particle size of approximately 100 nm. To further assess the particle size of ADSC-Exos, we employed nanoflow cytometry. The results from nanoparticle tracking analysis (NTA), depicted in Figure 1B, indicated that the particle size of ADSCs-Exos measured 81.43 nm. Next, we evaluated exosome-specific markers. As shown in Figure 1C, TSG101 was present in both total cellular protein and exosomes. In contrast, calnexin was detected in total protein but was not detected in the exosomes. This finding suggests that TSG101 is specifically expressed in exosomes and is not the result of contamination due to cellular protein leakage during the purification process. These results confirmed that we successfully purified highly pure ADSC-Exos. We subsequently co-incubated ADSC-Exos with NPP-WSM and underwent another purification process to produce engineered exosomes, designated as NPP-WSM-Exos. We then characterized these exosomes. As shown in Figure 1D, the NPP-WSM-Exos retained their characteristic cup-shaped morphology and exhibited a particle size of approximately 100 nm. Further analysis of exosome size via nanoflow cytometry revealed a particle size of 87.9 nm for the purified exosomes, as shown in Figure 1E. As indicated in Figure 1F, compared to the total protein from ADSCs, NPP-WSM-Exos expressed TSG101 while remaining negative for calnexin. These findings suggest that NPP-WSM-Exos maintain their exosomal characteristics, with the observed increase in particle size likely attributed to the encapsulation of NPP-WSM.

### Compared with NPP-WSM, NPP-WSM-Exos can promote the proliferation of MC3T3 cells

**Figure 2.**
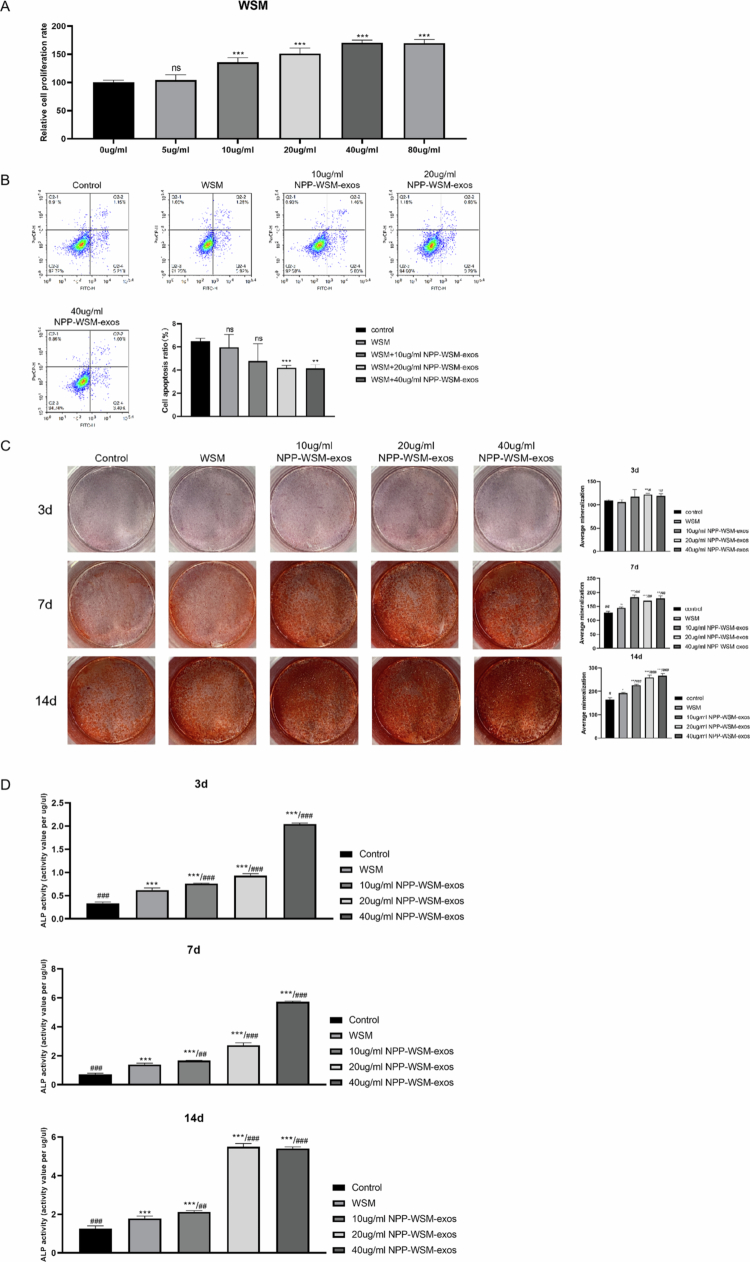
(A) CCK8 assay detects the effects of different concentrations of WSM on the viability of MC3T3 cells. (B) Flow cytometry was used to detect the effects of different concentrations of NPP-WSM-Exos and WSM on the apoptosis level of MC3T3 cells. (C) Alizarin red staining was used to detect the effects of different concentrations of NPP-WSM-Exos and WSM on the osteogenic differentiation ability of MC3T3 cells. (D) Biochemical experiments were conducted to detect the effects of different concentrations of NPP-WSM-Exos and WSM on alkaline phosphatase activity in MC3T3 cells. *p < 0.05, **p < 0.01, and ***p < 0.001.

The effect of NPP-WSM on MC3T3-E1 cell viability was evaluated using the CCK8 assay. As shown in Figure 2A, NPP-WSM promoted cell viability in a concentration-dependent manner within the range of 10–40 μg/mL. To assess the stimulatory effect of NPP-WSM-derived exosomes, 40 μg/mL NPP-WSM was used as a positive control, while NPP-WSM-Exos at 10, 20, and 40 μg/mL were designated as low-, medium-, and high-dose experimental groups, respectively. Figure 2B indicates that apoptosis levels in the NPP-WSM group (5.96 ± 1.1%) and the 10 μg/mL group (4.77 ± 1.49%) were not significantly different from that in the control group (6.49 ± 0.25%). Both the 20 μg/mL group (4.2 ± 0.2%, *p* < 0.001) and the 40 μg/mL group (4.13 ± 0.32, *p* < 0.01) significantly reduced apoptosis in MC3T3-E1 cells. Alizarin Red staining (Figure 2C) and an ALP activity assay (Figure 2D) demonstrated the osteogenic differentiation capacity of MC3T3-E1 cells under various stimulation conditions and time points. On day 3, no significant difference in calcification was observed between the WSM group (OD = 106.3 ± 5.03) and the control group (OD = 109.04 ± 2.29). The 10 μg/mL group (OD = 112.54 ± 14.09) also showed no significant difference compared to the control and WSM groups. The 20 μg/mL group (OD = 122.31 ± 2.32) exhibited significantly higher calcification than the control and WSM groups (*p* < 0.01), and the 40 μg/mL group (OD = 119.1 ± 4.63) also showed a significant increase (*p* < 0.05). On day 7, the WSM group (OD = 144.97 ± 2.74) showed significantly greater calcification than the control group (OD = 128.4 ± 3.9; *p* < 0.05). The 10 μg/mL group (OD = 183.66 ± 7.17) showed significantly higher calcification than both the control (*p* < 0.001) and WSM groups (*p* < 0.01). The 20 μg/mL group (OD = 171.24 ± 3.84) and the 40 μg/mL group (OD = 179.23 ± 7.8) also exhibited significantly higher calcification compared to the control (*p* < 0.001 and *p* < 0.01, respectively) and WSM groups (*p* < 0.01 for both). On day 3, the alkaline phosphatase activity in the WSM group (0.2 ± 0.02 U/μg protein) was not significantly different from that in the control group (0.23 ± 0.01 U/μg protein). The 10 μg/mL (0.22 ± 0.01 U/μg protein) and 20 Ug/mL (0.22 ± 0.004 U/μg protein) groups also showed no significant difference compared to the control and WSM groups. The 40 μg/mL group (0.23 ± 0.01 U/μg protein) demonstrated a significant increase compared to the control (*p* < 0.05). On day 7, the WSM group (0.36 ± 0.05 U/μg protein) showed significantly higher alkaline phosphatase activity than the control group (0.28 ± 0.04 U/μg protein, *p* < 0.01). The 10 μg/mL (0.34 ± 0.01 U/μg protein), 20 μg/mL (0.33 ± 0.01 U/μg protein), and 40 μg/mL (0.38 ± 0.02 U/μg protein) groups all exhibited significantly higher activity than the control group (*p* < 0.05, *p* < 0.05, and *p* < 0.01, respectively). On day 14, the WSM group (0.61 ± 0.003 U/μg protein) showed significantly higher alkaline phosphatase activity than the control group (0.5 ± 0.05 U/μg protein, *p* < 0.05). The 10 μg/mL (0.82 ± 0.05 U/μg protein), 20 μg/mL (0.88 ± 0.08 U/μg protein), and 40 μg/mL (0.88 ± 0.04 U/μg protein) groups all demonstrated significantly higher activity than the control group (*p* < 0.001 for all).

### NPP-WSM-Exos promote the expression of osteogenesis-related proteins more effectively than NPP-WSM

**Figures 3.**
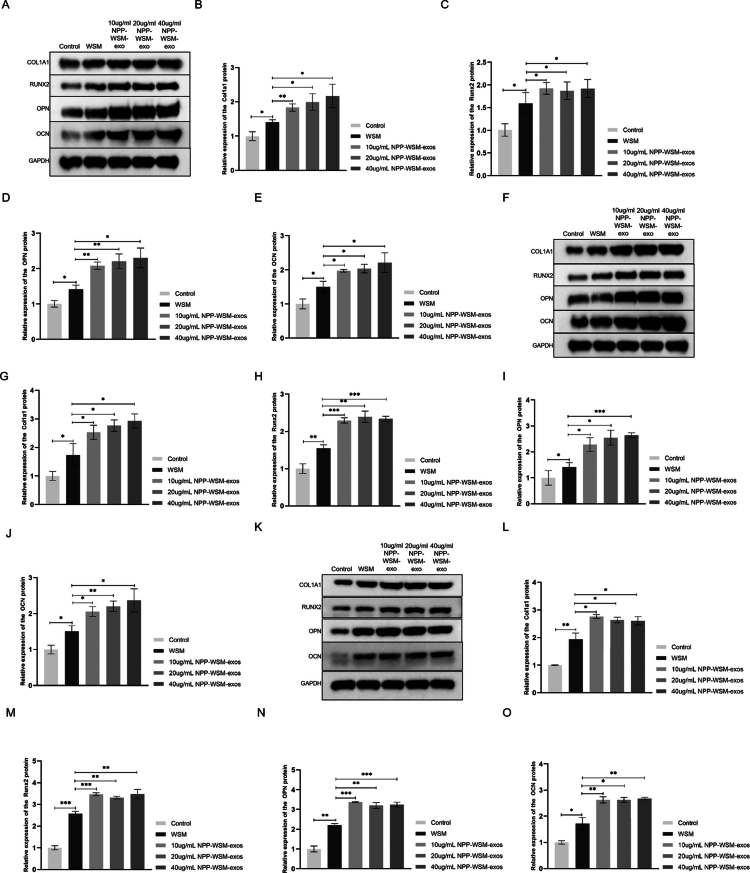
(A–E) Western blot analysis of the expression levels of osteogenic differentiation-related proteins in different groups on day 3 (A) and statistical graphs (B: COLA1A1, C: RUNX2, D: OPN, and E: OCN). (F–J) Western blot analysis of the expression levels of osteogenic differentiation-related proteins in different groups on day 7 (F) and statistical graphs (G: COLA1A1, H: RUNX2, I: OPN, and J: OCN). (K–O) Western blot analysis of the expression levels of osteogenic differentiation-related proteins in different groups on day 14 (K) and statistical graphs (L: COLA1A1, M: RUNX2, N: OPN, and O: OCN). *p < 0.05, **p < 0.01, and ***p<0.001.

We further examined the effects of NPP-WSM-Exos on the expression levels of osteogenesis-related genes. As shown in Figures 3A–E, on day 3, compared with those in the control group, the expression levels of COL1A1, RUNX2, OCN, and OPN in the WSM group cells were significantly upregulated by 1.4-fold (*p* < 0.05), 1.59-fold (*p* < 0.05), 1.49-fold (*p *< 0.05), and 1.41-fold (*p* < 0.05), respectively, and the above indicators are statistically significant. Administration of 10 µg/mL NPP-WSM-Exos led to a 1.83-fold increase in COL1A1 expression (*p* < 0.01), a 2.1-fold increase in RUNX2 (*p *< 0.05), a 1.97-fold increase in OCN (*p* < 0.01), and a 2.08-fold increase in OPN (*p* < 0.05) compared to the WSM group. All of these indicators demonstrated statistically significant differences. Administration of 20 µg/mL NPP-WSM-Exos resulted in 1.99-fold, 2.05-fold, 2.03-fold, and 2.21-fold increases in the expression of COL1A1, RUNX2, OCN, and OPN, respectively, compared to the WSM group (*p* < 0.05 for COL1A1, RUNX2, and OPN; *p* < 0.01 for OCN). These increases were statistically significant. Administration of 40 µg/mL NPP-WSM-Exos increased the expression of COL1A1 by 2.17-fold (*p* < 0.05), RUNX2 by 2.09-fold (*p* < 0.05), OCN by 2.21-fold (*p* < 0.05), and OPN by 2.3-fold (*p* < 0.05). All of these markers demonstrated statistically significant differences compared to the WSM group.

Figure 3F‒J demonstrates that, on day 7, the WSM group showed significant upregulation of COL1A1 (1.77-fold, *p* < 0.05), RUNX2 (1.56-fold, *p* < 0.05), OCN (1.50-fold, *p* < 0.05), and OPN (1.66-fold, *p* < 0.05) compared to the control group. All of these indicators also exhibited statistically significant differences when compared to the WSM group. In the 10 µg/mL NPP-WSM-Exos group, the expression levels of COL1A1, RUNX2, OCN, and OPN were upregulated by 2.53-fold (*p* < 0.05), 2.29-fold (*p* < 0.001), 2.05-fold (*p* < 0.05), and 2.28-fold (*p* < 0.05), respectively, compared to the WSM group. All of the above indicators showed statistically significant differences. Treatment with 20 µg/mL NPP-WSM-Exos increased COL1A1 expression by 2.76-fold (*p* < 0.05), RUNX2 by 2.39-fold (*p* < 0.01), OCN by 2.2-fold (*p* < 0.05), and OPN by 2.54-fold (*p* < 0.01), respectively, compared to the WSM group. All of the above indicators showed statistically significant differences. Meanwhile, 40 µg/mL NPP-WSM-Exos treatment led to the upregulation of COL1A1 by 2.92-fold (*p* < 0.05), RUNX2 by 2.33-fold (*p* < 0.001), OCN by 2.36-fold (*p* < 0.001), and OPN by 2.64-fold (*p* < 0.05) compared to the WSM group. All of the above indicators showed statistically significant differences.

On day 14 (Figure 3F‒J), the WSM group exhibited increased expression of COL1A1 by 1.77-fold (*p *< 0.01), RUNX2 by 1.56-fold (*p* < 0.001), OCN by 1.50-fold (*p* < 0.01), and OPN by 1.66-fold (*p* < 0.01) compared to the control group. All the indicators demonstrated statistically significant differences. The 10 µg/mL NPP-WSM-Exos treatment group showed upregulation of COL1A1 by 2.53-fold (*p* < 0.05), RUNX2 by 2.29-fold (*p* < 0.001), OCN by 2.05-fold (*p* < 0.001), and OPN by 2.28-fold (*p* < 0.01) compared to the WSM group. All changes were statistically significant. In the 20 µg/mL NPP-WSM-Exos group, the expression levels of COL1A1, RUNX2, OCN, and OPN increased by 2.76-fold (*p* < 0.05), 2.39-fold (*p* < 0.01), 2.2-fold (*p* < 0.01), and 2.54-fold (*p* < 0.05), respectively, compared to the WSM group. These differences were statistically significant. Administration of 40 µg/mL NPP-WSM-Exos increased COL1A1 by 2.92-fold (*p* < 0.05), RUNX2 by 2.33-fold (*p* < 0.01), OCN by 2.36-fold (*p* < 0.001), and OPN by 2.64-fold (*p* < 0.01) compared to the WSM group. All differences were statistically significant.

Taken together, these results demonstrate that NPP-WSM-Exos can more effectively promote the osteogenic differentiation of MC3T3-E1 cells.

### Transcriptome analysis revealed that NPP-WSM-Exos uniquely activated the PI3K/AKT pathway

**Figure 4.**
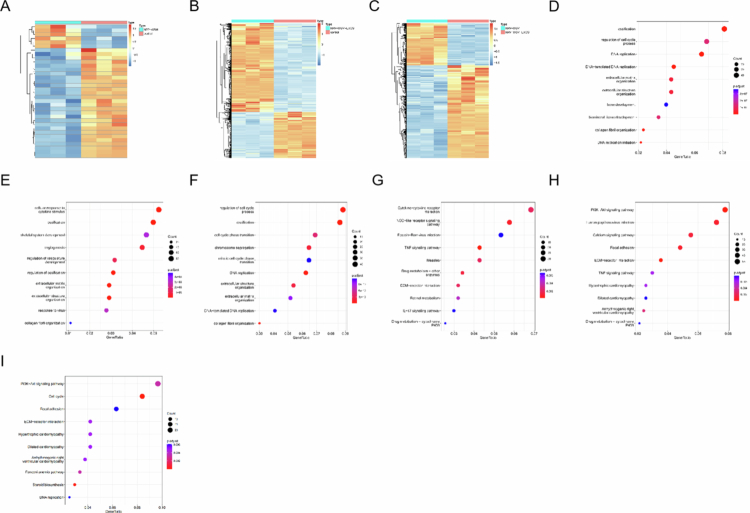
(A–C) Cluster heatmap showing the differences in transcriptome expression levels among the different groups (A: NPP-WSM vs control, B: NPP-WSM-Exos vs control, and C: NPP-WSM vs NPP-WSM-Exos). (D–F) GO analysis showed functional differences among the different groups (D: NPP-WSM vs control, E: NPP-WSM-Exos vs control, and F: NPP-WSM vs NPP-WSM-Exos). (G–I) KEGG analysis showed functional differences among different groups (G: NPP-WSM vs control, H: NPP-WSM-Exos vs control, and I: NPP-WSM vs NPP-WSM-Exos).

As shown in Figure 4A–C, hierarchical clustering revealed clear transcriptomic differences among the control, WSM, and NPP-WSM-Exos groups, with significant divergence also observed between the WSM and NPP-WSM-Exos groups. Gene Ontology (GO) enrichment analysis (Figure 4D–F) indicated that, relative to the control group, both the WSM and NPP-WSM-Exos treatments were significantly enriched in the biological process “ossification,” suggesting that both interventions promote osteogenic differentiation of MC3T3 cells at the transcriptomic level. Notably, when compared with the WSM group, differentially expressed genes in the NPP-WSM-Exos group were significantly enriched in “regulation of the cell cycle process,” indicating that NPP-WSM-Exos not only enhance osteogenic differentiation but also stimulate MC3T3 cell proliferation, thereby improving osteogenic efficiency.

We further explored the signaling pathways underlying these effects. As shown in Figure 4G, WSM treatment activated the cytokine–cytokine receptor interaction pathway, suggesting that trace proteins in WSM interact with MC3T3 surface receptors to trigger downstream signaling and facilitate osteogenic differentiation. In contrast, NPP-WSM-Exos treatment (Figure 4H) not only activated the PI3K/AKT pathway, which drives proliferation, but also activated the ECM–receptor interaction pathway, similar to WSM, by engaging cell surface receptors to promote osteogenesis. Importantly, direct comparison with the WSM group (Figure 4I) revealed that the activation of PI3K/AKT signaling was specific to NPP-WSM-Exos, underscoring that this proliferative effect is uniquely conferred by the exosomes rather than by WSM alone.

**Figure 5.**
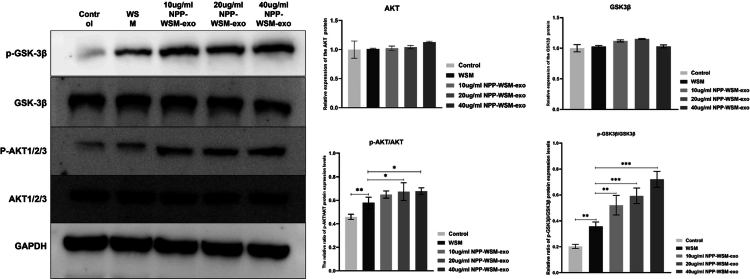
Western blot analysis was performed to verify the expression and phosphorylation levels of the target protein in each group of cells.

It is known that AKT activation inhibits GSK-3β activity by increasing the phosphorylation level of GSK-3β at the Ser9 site, thereby promoting β-catenin nuclear translocation and activating the osteogenic differentiation pathway. Therefore, does NPP-WSM-Exos promote osteogenic differentiation by inhibiting GSK-3β activity? We performed Western blot experiments to detect protein levels, as shown in Figure 5. There were no significant differences in the total protein expression levels of AKT and GSK3β among the groups. However, compared to the control group, the phosphorylation level of AKT protein in the WSM group significantly increased from 45.93 ± 2.24% to 57.92 ± 4.7%, *p* < 0.01, and the phosphorylation level of GSK3β Ser9 site significantly increased from 20.33 ± 1.58% to 35.79 ± 3.33%, *p* < 0.001. Compared to the WSM group, the AKT protein phosphorylation level in the 10 µg/mL group significantly increased to 64.89 ± 3.05%, *p* < 0.001, and the GSK3β Ser9 site phosphorylation level significantly increased to 59.33 ± 6.01%, *p* < 0.001. Compared to the WSM group, the AKT protein phosphorylation level in the 20 µg/mL group significantly increased to 67.37 ± 7.51%, *p* < 0.001, and the GSK3β Ser9 site phosphorylation level significantly increased to 59.33 ± 6.01%, *p* < 0.001. Compared to the WSM group, the AKT protein phosphorylation level in the 40 µg/mL group significantly increased to 67.71 ± 2.85%, *p *< 0.001, and the GSK3β Ser9 site phosphorylation level significantly increased to 72.07 ± 6.12%, *p* < 0.001. These Western blot results indicate that the NPP-WSM-Exos group significantly increased the AKT-related pathway, thereby inhibiting GSK-3β activity and promoting β-catenin nuclear translocation to activate the osteogenic differentiation pathway. WSM also has the effect of inhibiting GSK-3β activity.

## Discussion

In the research of bone repair materials, hydrogels have become an ideal carrier for carrying active components (such as antibiotics or traditional Chinese medicine monomers) because of their good biocompatibility, tunable physicochemical properties, and three-dimensional biomimetic structure, achieving significant results. Existing research shows that hydrogel systems loaded with vancomycin or anti-inflammatory traditional Chinese medicine monomers (such as baicalin) can effectively inhibit bacterial infection and regulate inflammatory factor levels through local sustained release and have initially shown the ability to promote macrophage polarization towards the M2 phenotype, thus creating a relatively favorable microenvironment for bone regeneration. However, this strategy has inherent limitations in its mechanism of action: it essentially relies on the pharmacological effects of the loaded chemical drugs for “passive intervention,” resulting in relatively singular and indirect regulation of complex biological processes, making it difficult to achieve precise synergy between the immune microenvironment and the regenerative process.

In contrast, exosomes, as a novel natural nanocarrier, represent a shift from the concept of “drug loading in inert materials” to “active programming of bioactive signals.” The key difference between exosomes and hydrogels lies in the fact that exosomes are not simply physical encapsulation platforms but rather signaling complexes secreted by source cells, which are rich in bioactive molecules such as proteins, nucleic acids, and lipids. This gives them fundamental advantages: first, the precision and endogenous nature of their mechanism of action. Exosomes can actively deliver their carried functional miRNAs (such as miR-223 and miR-146a) and cytokines to target cells (such as macrophages and mesenchymal stem cells) through surface ligand fusion with membranes, directly regulating key signaling pathways such as the STAT6 and AKT. This allows them to more efficiently and specifically drive macrophages toward reparative M2 polarization and simultaneously activate the osteogenic differentiation of stem cells. Second, the pluripotency and synergistic effects of their functions. A single exosome formulation can simultaneously achieve multiple biological effects, including immunomodulation, angiogenesis, and direct osteogenic effects. This “integrated” function is difficult to achieve with simple drug-loaded systems. Therefore, exosome-based materials transcend the “alternative therapy” logic of traditional hydrogel systems, which primarily rely on drug release. Instead, they shift toward “programming” the damaged microenvironment by reshaping intercellular communication networks, providing a revolutionary strategy for developing next-generation intelligent, biomimetic bone repair materials. Future research may focus on combining engineered exosomes with hydrogels that provide structural support to construct composite material systems that combine the precise release of biological signals with ideal mechanical properties.

Using exosome-based delivery systems to encapsulate bioactive compounds while maintaining their integrity is a current research hotspot and a key focus in the biopharmaceutical field.^19^ Numerous studies have demonstrated the feasibility of this approach, but it also presents numerous challenges. The core challenge lies in efficiently loading drugs and other bioactive molecules into exosomes while maximally maintaining the integrity and bioactivity of the exosome's native membrane.^20^^,^^21^ Currently, researchers have developed a variety of loading techniques, which are categorized primarily as endogenous loading (intervention prior to exosome production) and exogenous loading (loading of exosomes after isolation). Exogenous methods such as electroporation, sonication, and extrusion are widely used because of their straightforward operation, but these methods carry the risk of disrupting exosome membrane integrity.^22^ Therefore, academic research has focused on developing gentler and more efficient loading methods and optimizing existing technologies to advance exosome-based drug delivery systems toward clinical application. In this study, we successfully encapsulated NPP-WSM with exosomes derived from ADSCs, generating NPP-WSM-Exos. Detailed characterization confirmed that these engineered exosomes retained typical exosomal morphology and specific markers while exhibiting a slight increase in particle size, likely due to the incorporation of NPP-WSM. These findings demonstrate the feasibility of utilizing exosome-based delivery systems to encapsulate bioactive compounds while maintaining exosome integrity.

Functionally, our results demonstrated that both WSM and NPP-WSM-Exos significantly promoted osteogenic differentiation in MC3T3 cells, as evidenced by enhanced alkaline phosphatase activity, increased calcification, and transcriptome enrichment in osteogenesis-related biological processes. Importantly, compared with WSM, NPP-WSM-Exos exhibited superior efficacy, not only in enhancing osteogenic differentiation but also in stimulating MC3T3 cell proliferation. This dual effect of NPP-WSM-Exos provides a mechanistic explanation for their superior efficacy in promoting bone formation.

Pathway analysis further elucidated the molecular basis for these effects. WSM alone activated the cytokine‒cytokine receptor interaction pathway, suggesting that trace proteins interact with cell surface receptors and trigger downstream signaling. In contrast, NPP-WSM-Exos activated both the extracellular matrix (ECM)–receptor interaction pathway and the PI3K/AKT signaling pathway. The ECM–receptor pathway supports osteogenesis through cell‒matrix interactions, while the PI3K/AKT signaling pathway is a well-established driver of proliferation and survival.^23-25^ Pathway analysis further clarified the molecular mechanisms underlying these effects. WSM alone activated the cytokine‒cytokine receptor signaling pathway, suggesting that trace proteins interact with cell–surface receptors and initiate downstream signaling. In contrast, NPP-WSM-Exos activated both the extracellular matrix (ECM)–receptor interaction pathway and the PI3K/AKT signaling pathway. AKT inhibits GSK-3β activity by increasing the phosphorylation of Ser9, thereby promoting β-catenin nuclear translocation and activating osteogenic differentiation pathways. The experimental results confirmed that NPP-WSM-Exos effectively inhibited GSK-3β phosphorylation at Ser9. These findings indicate that specific activation of the PI3K/AKT signaling pathway in the NPP-WSM-Exos group underscores the unique functional advantages conferred by exosome encapsulation, potentially contributing to synergistic enhancement of proliferation and osteogenic differentiation.

These findings have several important implications. Exosome-based delivery of bioactive compounds offers potential to overcome the limitations of direct drug delivery by improving drug stability, cellular uptake, and functional targeting. Additionally, NPP-WSM-Exos can simultaneously promote cell proliferation and differentiation, indicating their potential application in bone tissue engineering and regenerative medicine, particularly for conditions such as osteoporosis and fracture repair, where both processes are essential.

However, this study has certain limitations. Mechanistic conclusions were supported only by omics analysis and pathway-target gene expression levels; animal studies and cell function recovery experiments were not performed. Future research should investigate the biodistribution, pharmacokinetics, and long-term safety of NPP-WSM-Exos to better assess their translational potential.

In summary, NPP-WSM-Exos represent a novel engineered exosome platform that effectively enhances the osteogenic differentiation and proliferation of MC3T3 cells by activating extracellular matrix receptors and the AKT/GSK3β pathway. These results highlight the potential of engineered exosomes as a multifunctional delivery system for bone tissue engineering and their promise in advancing bone regeneration therapeutic strategies.

## Data Availability

The data can be obtained by sending an email to the corresponding author.
